# Effects of Music on Agitation in Dementia: A Meta-Analysis

**DOI:** 10.3389/fpsyg.2017.00742

**Published:** 2017-05-16

**Authors:** Siv K. A. Pedersen, Per N. Andersen, Ricardo G. Lugo, Marita Andreassen, Stefan Sütterlin

**Affiliations:** ^1^Department of Psychology, Inland Norway University of Applied SciencesLillehammer, Norway; ^2^Department of Education and Social Work, Inland Norway University of Applied SciencesLillehammer, Norway; ^3^Center for Clinical Neuroscience, Oslo University HospitalOslo, Norway

**Keywords:** music intervention, agitation, dementia, meta-analysis, therapy

## Abstract

Agitation is a common problem in patients suffering from dementia and encompasses a variety of behaviors such as repetitive acts, restlessness, wandering, and aggressive behaviors. Agitation reduces the probability of positive social interaction and increases the psychological and organizational burden. While medical interventions are common, there is need for complementary or alternative methods. Music intervention has been brought forward as a promising method to reduce agitation in dementia. While interventions, target groups and research designs differ, there has so far not been a systematic overview assessing the effect of music intervention for agitation in patients with dementia. A meta-analysis was conducted in order to investigate possible effects of music interventions. Twelve studies met inclusion criteria. Music intervention had a medium overall effect on agitation in dementia, suggesting robust clinical relevance. While the moderate number of studies does not allow for further differentiation between sub-types of music intervention, the sub-group comparisons indicated promising pathways for future systematic reviews. This meta-analysis is the first systematic and quantitative overview supporting clinically and statistically robust effects of music intervention on agitation in dementia. The analysis provides further arguments for this non-pharmacological approach and highlights needs for future systematic research reviews for the investigation of intervention types.

## Introduction

The American Music Therapy Association (AMTA) defines music therapy (MT) as “the clinical and evidence-based use of music interventions to accomplish individualized goals within a therapeutic relationship by a credentialed professional who has completed an approved music therapy program” (American Music Therapy Association, 2006). Other definitions underline MT's role more specifically as a therapeutic medium to address “developmental, adaptive, and rehabilitative goals in the areas of psychosocial, cognitive, and sensorimotor behavior of individuals with disabilities” (Hallam et al., 2009). Music therapy can be applied in a range of possible settings, such as “everyday environments with individuals, groups, families, or communities who seek to optimize their quality of life and improve their physical, social, communicative, emotional, intellectual, and spiritual health and well-being” (World Federation of Music Therapy, 2011). Clinical experiences and research results suggest MT to be an intervention with considerable positive health outcomes (MacDonald, 2013). The National Institute for Health and Care Excellence (NICE) guidelines for treating people with dementia who have comorbid agitation recommends a range of sensory stimulation interventions (National Institute for Clinical Excellence, 2006). These include aromatherapy, music intervention, animal-assisted therapy, massage, and multisensory stimulation (National Institute for Clinical Excellence, 2006). According to NICE, a range of health and social care staff and volunteers may deliver the interventions with appropriate training and supervision. Furthermore, the interventions should be tailored to the person's preferences, skills, abilities, and responsiveness of treatment.

For the purpose of this meta-analysis, music intervention was defined as the controlled use of music in a therapeutic setting to accomplish individualized goals within physiological, psychological, and emotional well-being during the treatment of an illness or disease.

Despite anecdotal evidence of positive effects, systematic research in controlled experimental settings aiming to quantify health outcomes following MI is still scarce. The heterogeneity of intervention types, target groups, and clinical context requires a systematic overview before firm conclusions on the specific effects of MI can be made.

One area in which MI has been repeatedly reported to promote positive outcomes is agitation in demented patients. Agitation *per se* is no diagnosis, but consists of a transdiagnostic group of symptoms that may reflect an underlying disorder and has been defined as “inappropriate verbal, vocal, or motor activity that is not judged by an outside observer to result directly from the needs or confusion of the agitated individual (p. 712)” (Cohen-Mansfield and Billing, 1986). The prevalence rates for agitation in dementia range from 20 to over 80% depending on the definition used and the means of assessment (Sourial et al., 2001). Agitation encompasses a variety of behaviors such as repetitive acts, restlessness, wandering, and aggressive behaviors toward oneself or others (Cohen-Mansfield, 2008). It is considered one of the core features of behavioral and psychological symptoms of dementia (BPSD) besides others such as anxiety, depression, irritability, hallucinations, delusions, and eating problems (Cerejeira et al., 2012). BPSD and particularly agitation pose an increased burden for both patients and caregivers and are common causes of hospitalization and institutionalization of people with dementia (Schulz and Williamson, 1991). Agitation has detrimental psychosocial consequences for the patient and reduces the probability of positive social interaction and thus cognitive stimulation. Typical treatment in severe depression often includes antidepressants and anxiolytic pharmaceuticals to control emotional symptoms, and antipsychotic drugs to control hallucinations, delusions, and are aimed to also reduce agitation (Caltagirone et al., 2005). Because of an increased risk of medication misuse, increased health care costs (Cerejeira et al., 2012), and a number of adverse side effects like nausea, insomnia, anorexia, gastrointestinal discomfort, and fatigue (Caltagirone et al., 2005), it has been recommended that non-pharmacological treatment is given first priority if the patient shows distressing behavioral symptoms (Azermai et al., 2011). The reason why pharmacological intervention is widely in use to reduce agitation in dementia is most likely due to a current lack of other proven effective treatments (Hansen et al., 2007).

### Musical interventions to reduce agitation

Musical interventions to reduce agitation can be administered as different interventions across several settings. Active MI involves the participants actively by means of singing, dancing or instrument playing. In passive MI, the patients listen to live or recorded music without being actively engaged. The music is either prescribed by the therapist without any prior knowledge of the patient's preferences, or it is selected in accordance with the patient's preferences. In individual MI, the patient experiences music alone or together with the therapist, whereas group MI refers to shared music experience between two or more patients.

Numerous studies indicated beneficial effects of MI on reducing agitation in people with dementia (Clark et al., 1998; Remington, 2002; Sung et al., 2006a; Raglio et al., 2008, 2010; Lin et al., 2011; Janata, 2012; Ridder et al., 2013; Sakamoto et al., 2013). One factor contributing to the occurrence and maintenance of agitation lies in impaired capabilities to communicate, such as to express one's emotions or desires verbally (Sung et al., 2006b). It has been argued that MI provides a channel for a more appropriate expression of emotions (Sung et al., 2006a) while other studies did not replicate positive effects of MI on agitation (Sung et al., 2006b, 2012; Raglio et al., 2015). A number of different therapy forms co-exists and have been investigated. Group MI provides those with dementia an opportunity for communication and social interaction which can divert attention away from environmental and emotional cues that can provoke agitation (Sung et al., 2006a, 2012; Raglio et al., 2010; Lin et al., 2011). Active MI act as a powerful stimulus that promotes socialization, involvement with the environment, and awareness (Raglio et al., 2008, 2010; Sakamoto et al., 2013). In addition to this, active MI is shown to reduce stress and apathy, and negative behaviors such as aggressiveness and agitation (Sakamoto et al., 2013) by helping the patients to create meaningful activities (Svansdottir and Snaedal, 2006). Passive music listening is reported to have a beneficial effect on agitated behavior by eliciting repressed feelings (Lin et al., 2011). Music based on the patient's preferences has been argued to have a good effect on agitation in dementia (Sung et al., 2006a,b; Raglio et al., 2008; Sakamoto et al., 2013). Familiar music with pleasing sound can possibly remind the listening patients of their lives before the outbreak of the disease and a life beyond the care facility (Lin et al., 2011). The use of individualized chosen has been argued to provide more arousing and positive emotional memories (El Haj et al., 2012), helping the patients to attain a state of calmness and greater relaxation (Lin et al., 2011), and consequentially alleviates agitated behavior. In a first systematic overview of 10 randomized controlled studies, Vink et al. (2004) investigated the effects of MI in treatment of behavioral, social, cognitive, and emotional problems of elderly people with dementia. The authors concluded that there was a high probability of positive effects on cognitive abilities, quality of life and agitation, but conceded that methodological shortcomings and inconsistencies in the published work available by 2004 reduced the robustness and comparability of the reported findings and limited any conclusions that could be drawn in regards to robust effects of MI intervention (Vink et al., 2004, 2011). Recent additional research with higher methodological standards has been published corroborating the assumption of clinically significant positive outcomes in BPSD, particularly for managing agitation and aggressiveness (e.g., Raglio et al., 2008).

A systematic overview and quantification of the reported effects and analysis of potential publication bias, however, is currently lacking. It remains so far unclear whether systematically controlled intervention studies support the notion of robust and clinically significant reduction of agitation in dementia, and whether different types of intervention differ in regards of their achieved clinical effects. This meta-analysis attempts to provide the first systematic review over controlled intervention studies to suggest a conclusion on the magnitude of effects. In addition, an evaluation of intervention characteristics such as group- vs. personal therapy, prescribed vs. individually chosen music, active vs. passive participation and the degree of dementia has been conducted to shed a light on which type of intervention might be most beneficial.

More precisely, this meta-analysis will (1) investigate whether music therapy is an effective intervention for reducing agitated behaviors in dementia, will (2) compare personalized and group interventions, will (3) assess the effect of music preference by comparing music based on the patient's preference with music without prior consulting of patients or caregivers, and will (4) compare benefits of active vs. passive music intervention.

## Methods

### Data collection

**Figure 1:**
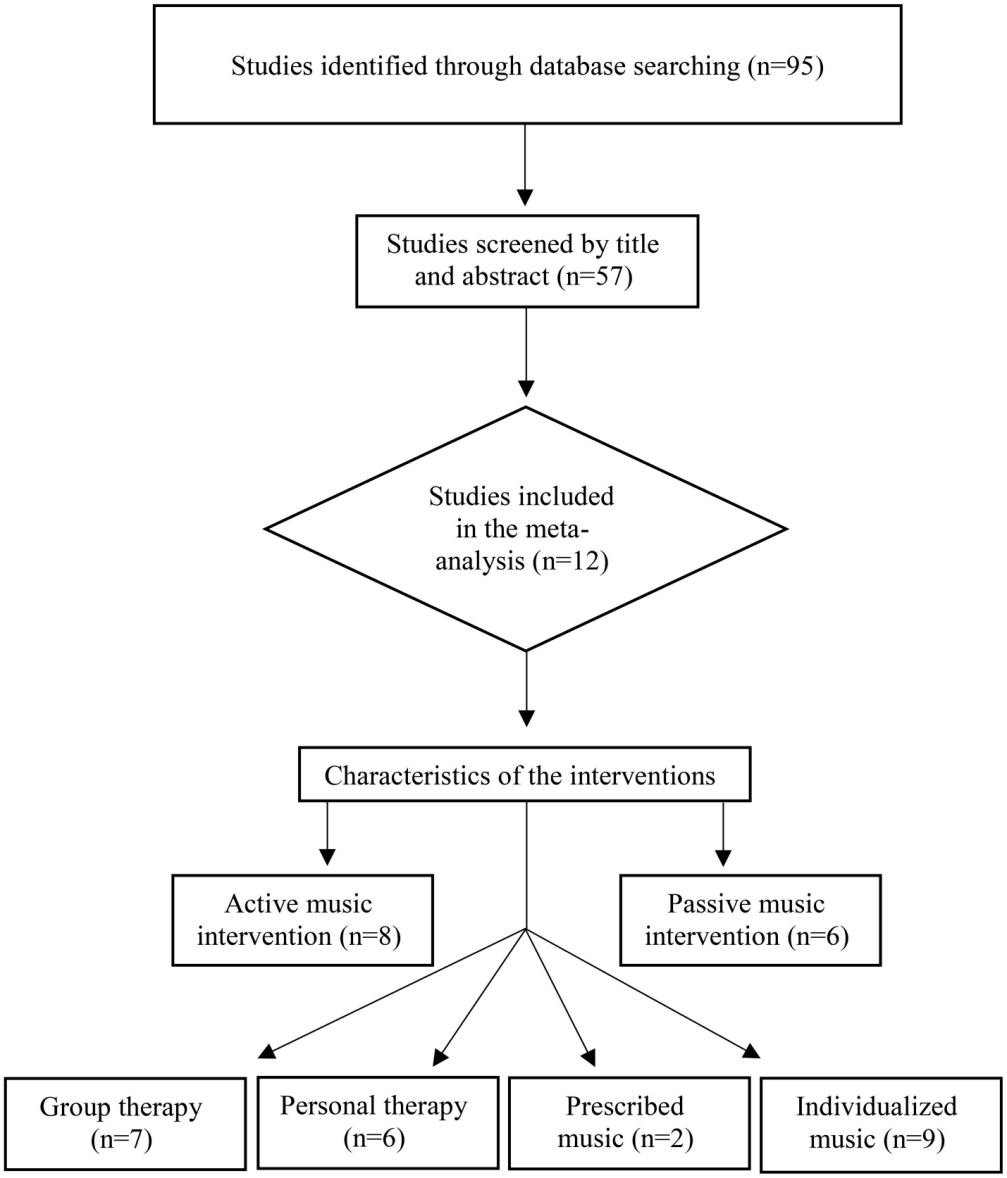
**Flow chart process of study selection and categorization**.

The studies included in the meta-analysis used to evaluate the effect of music intervention on agitation in demented people were identified via databases PsycINFO, PsycArticles, Medline/PubMed, CINAHL, and Academic Search Premier. Each database was searched by using the key terms *Music* AND *dementia, Music* AND *Alzheimer's* combined with *music therapy* OR *music intervention* AND *agitation* limited to English language. Because the use of music therapy as intervention for people with dementia is a relatively new field of study, no limitation for the year of publication was applied. All searches were made during October 2015 throughout February 2016. The search engine Google Scholar was also applied to increase the chance of research findings in gray literature (repositories, dissertations, etc.). In addition, a review of the references from previous overview articles was conducted. Figure 1 shows the selection of articles included in the later meta-analysis.

### Inclusion and exclusion criteria

This meta-analysis included original research fulfilling the following inclusion criteria: (1) Used randomized controlled study as design. (2) The study population consisted exclusively of elderly individuals who were formally diagnosed in accordance with the Diagnostic and Statistical Manual of Mental Disorders or International Classification of Diseases V. In addition, other accepted clinical criteria like Global Deterioration Scale (GDS) used to examine the progression of Alzheimer's disease (Reisberg et al., 1982), and the Clinical Dementia Rating (CDR) or Mini-Mental State Examination (MMSE) were accepted as inclusion criteria. (3) The study used a music intervention aiming at reducing agitation. (4) Agitation was assessed by validated and reliable scales. (5) The study provided effect sizes or reported quantitative data sufficient for calculation of effect sizes. (6) Data were available from both pre- and post-intervention time points. (7) The presence of a trained music therapist as required for the MT definition was not required in this meta-analysis.

Exclusion criteria were as follows: (1) Studies with <5 participants. (2) Uncontrolled studies.

### Intervention outcome measures

The instruments used in the studies to measure agitation include the Cohen-Mansfield Agitation Inventory (CMAI; Cohen-Mansfield et al., 1989) assessing agitated behaviors and their frequencies, Neuropsychiatric Inventory (NPI; Cummings et al., 1994), the Behavioral Pathology in Alzheimer's Disease Rating Scale (BEHAVE-AD; Reisberg et al., 1987) and an agitation checklist. The CMAI is administered by trained research staff or caregivers. Interrater reliability (Cronbach's alpha) of the CMAI has been reported between 0.82 and 0.93 across different ethnic populations (Finkel et al., 1992). The NPI is an inventory used in the studies in order to obtain information about the patient's psychopathology, in particular agitation, for the purpose of the included studies. The NPI is based on responses from an informed observer or caregiver. Internal consistency reliability for the NPI is reported with a Cronbach's alpha of 0.88 (Cummings et al., 1994). BEHAVE-AD is based on an interview of an informant who knows the patient. Interrater reliability (Cronbach's alpha) of the BEHAVE-AD has been reported to be 0.96 (Reisberg et al., 1996). The study of Clark et al. used an agitation checklist developed for the purpose of this particular study (Clark et al., 1998). The target behavior included hitting, biting, screaming, crying, abusive language, wandering, spitting, refusals to cooperate, pinching, scratching, kicking, throwing of objects, and grabbing. The authors reported an internal consistency of Cronbach's alpha = 0.90 after documenting their reporting's at the beginning of their study and at approximately 2-week intervals during data collection (Clark et al., 1998).

### Statistical analysis

#### Effect size

Excel 2013 and the Comprehensive Meta-Analysis software program version 2.0 were used to conduct the meta-analysis. Weighted average Cohen's *d* was used as overall effect size. For the individual studies, we computed *d* according to Morris (2008) as *d* = (M_*T*_*PRE*_ − M_*T*_*POST*_) − (M_*C*_*PRE*_ − M_*C*_*POST*_)/SD_*PRE*_ where M_*T*_*PRE*_ and M_*TPOST*_ are the pre and post mean scores for the treatment group, M_*C*_*PRE*_ and M_*C*_*POST*_ the mean scores for the control group, and SD_*PRE*_ is the pooled standard deviation for the pre-scores of both groups. Individual studies Significance level was set to alpha = 0.05. According to Cohen's criteria, an effect size of 0.2–0.4 reflects a low effect size, 0.5–0.7 moderate effect size, and >0.8 is considered a large effect size (Cohen, 1992). These criteria should only be applied in the absence of better scientific criteria based on the research literature and are therefore applied here in the context of this new field of research where systematic studies are scarce. The random effects model was used in the data analysis.

#### Heterogeneity

Methodical differences in design, participants, interventions, or exposures increase the heterogeneity between studies and can partially account for observed differences in the results of included studies and thus limit the interpretational value of summarized findings. To ensure sufficient consistency of combined studies and conclude on the generalizability of the summarized findings, the *I*^2^ index of heterogeneity was calculated (Higgins et al., 2003). *I*^2^values of 0–25% were interpreted as no heterogeneity, 25–50% as representing low heterogeneity, 50–75% as moderate heterogeneity, and 75–100% as representing high heterogeneity between studies.

#### Publication bias

Publication bias reflects the fact that studies supporting the hypothesis are more likely to be published than null results (Easterbrook et al., 1991; Rothstein et al., 2006). A funnel plot and calculation of the Fail-Safe-N (the number of unpublished studies with mean effect of zero necessary to equalize the statistically significant effect in published studies) was completed (Egger et al., 1997).

## Results

### Study identification

An initial literature search using the chosen key terms identified 57 articles out of which 45 did not meet the inclusion criteria (see Figure 1). This included lack of quantitative data required for the calculation of effect sizes, inclusion of other diagnoses than dementia, review articles, and discussion papers. The remaining 12 articles were composed of randomized controlled trials (RCT) fulfilling minimum standards and were included in the meta-analysis. Figure 1 illustrates the process of study selection and categorization.

### Characteristics of the studies

Methodological main characteristics of included studies are summarized in Table 1.

**Table 1:** **Description of included studies**.

**Study and Year**	***N***	**Dementia severity**	**Intervention**	**Outcome measures**	**Group vs. individual therapy**	**Prescribed vs. individualized music**	**Active vs. passive music intervention**
Clark et al., 1998	Total *N*: 18 T/C: 9/9	Severe (MMSE)	Experimental group: 10 baths with favorite music during bathing, distributed on a 2 weeks period	Agitation checklist	Individual	Individualized	Passive
			Control group: Standard care (no music during bathing)				
Janata, 2012	Total *N*: 38 T/C: 19/19	Moderate to severe (MMSE)	Experimental group: session length (min): 25/65; Frequency (times/week): 84/12; Music streaming for several hours per day (two times in the morning and two times in the evening) each day for 12 weeks	CMAI	Individual	Individualized	Passive
			Control group: Standard care				
Lin et al., 2011	Total *N*: 100 T/C: 49/51	Moderate to severe (MMSE)	Experimental group: session length (min): 30; Frequency (times/week): 12/6; Two times a week for 6 weeks	MMSE	Group	Individualized	Passive
			Control group: Standard care				
Raglio et al., 2008	Total *N*: 59 T/C: 30/29	Moderate to severe (CDR)	Experimental group: session length (min): 30; Frequency (times/week): 30/16; Three cycles of 10 MT sessions within a period of 16 weeks	NPI	Group	Individualized	Active
			Control group: standard care				
Raglio et al., 2010	Total *N*: 60 T/C: 30/30	Severe (MMSE)	Experimental group: session length (min): 30; Frequency (times/week): 36/12; Three cycles of 12MT sessions each, Three times a week for 12 weeks	NPI	Group	Not mentioned	Active
			Control group: standard care				
Raglio et al., 2015	Total *N*: 120 T/T/C: 31/32/35	Moderate to severe (CDR and MMSE)	Experimental group: session length (min): 30; Frequency (times/week): 20/10; Twice a week for 10 weeks	NPI	Individual and group	Individualized	Active and passive
			Control group: Standard care				
Remington, 2002	Total *N*: 34 T/C: 17/17	Mild to severe	Four groups were used to test the effect of a 10 min exposure to either calming music, hand massage, or calming music and hand massage simultaneously, or no intervention (control). Only data from the music group and control group were used in the analysis.	CMAI	Individual	Prescribed	Passive
			Experimental group: 1 session with 10 min music intervention				
			Control group: standard care				
Ridder et al., 2013	Total *N*: 42 T/C: 21/21	Moderate to severe (MMSE and GDS)	Experimental group: session length (min): 30; Frequency (times/week): 12/6; Two times a week for 6 weeks	CMAI	Individual	Prescribed	Active
			Control group: standard care				
Sakamoto et al., 2013	Total *N*: 39 T/C: 13/13/13	Severe (MMSE)	Experimental group: session length (min): 30; Frequency (times/week): 10/10; Once a week for 10 weeks	BEHAVE-AD	Individual	Individualized	Active and passive
			Control group: standard care				
Sung et al., 2006a	Total *N*: 57 T/C: 32/25	Moderate to severe (GDS)	Experimental group: session length (min): 30; Frequency (times/week): 12/6; Two times a week for 6 weeks	CMAI	Group	Individualized	Active
			Control group: standard care				
Sung et al., 2006b	Total *N*: 36 T/C: 18/18	Moderate to severe (GDS)	Experimental group: session length (min): 30; Frequency (times/week): 8/4; Two times a week for 4 weeks	CMAI	Group	Individualized	Active
			Control group: standard care				
Sung et al., 2012	Total *N*: 55 T/C: 27/28	Mild to moderate (MMSE)	Experimental group: session length (min): 30; Frequency (times/week): 12/6; Two times a week for 6 weeks	CMAI	Group	Individualized	Active
			Control group: standard care				

*T/C, treatment/control group; MMSE, mini mental state examination; CDR, clinical dementia rating; GDS, global deterioration scale*.

#### Participants

A total of *N* = 658 participants were involved in the included 12 studies (*M* = 55, min = 9, max = 51). Five of the studies included only people with Alzheimer's disease; four studies included people with Alzheimer's disease, vascular dementia, mixed type dementia, Lewy body dementia, and frontotemporal dementia. The remaining three studies did not specify type of dementia. The degree of severity ranged from mild to severe.

#### Intervention

All but one of the studies used participants' preferred music in the interventions. The studies included in the meta-analysis varied in the use of practitioners carrying out interventions. Six of the studies used authorized music therapists. Five of the included studies used researchers or nurses who had completed music therapy courses. Another study used care workers that administered the music, while a trained music therapist analyzed the videotapes after the sessions. Only one study did not specify the type of practitioner used. The interventions took many different forms. Eight studies used a combination of methods, including singing, stretching, and clapping (active music intervention). Six studies used passive intervention therapy as intervention context. The music interventions in the different studies were administered through different forms, including music listening through headphones, CD players or live performances.

#### Overall effects of music intervention on agitation

**Figure 2:**
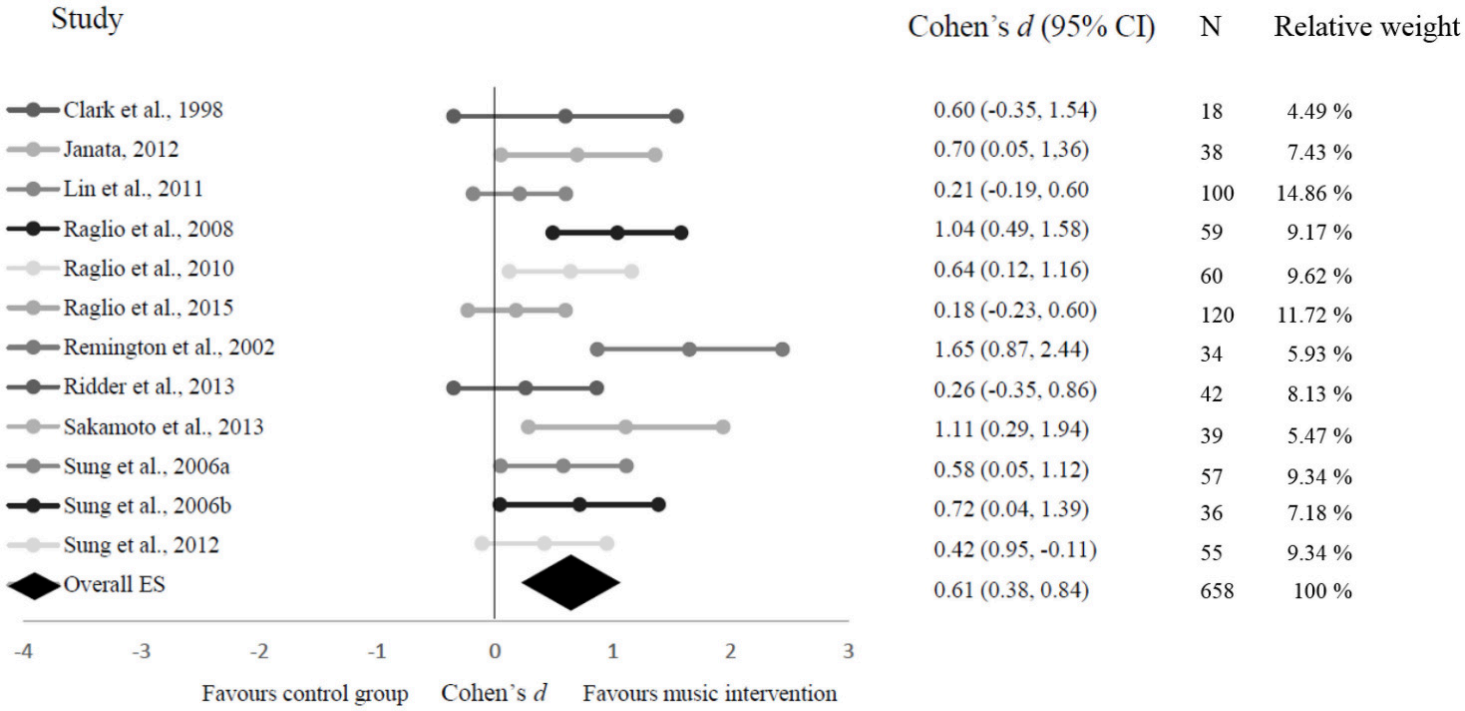
**Forest plot on the effect of music intervention in demented people**. The center point reflects the mean effect size between a confidence interval of 95%.

The first research question was whether music intervention is an effective intervention for reducing agitated behaviors in dementia. The effect sizes of music intervention in agitation are presented in Figure 2. The obtained mean effect size of the 12 studies included in this meta-analysis for exploring the effectiveness of music intervention on agitation was *d* = 0.61 with a 95% confidence interval (CI) of 0.38–0.84. The results indicate that music intervention significantly reduces agitated behaviors in demented people. According to common classification standards, a low-to-moderate heterogeneity was found between the 12 included studies (*Q* = 20.35; *p* = 0.001; *I*^2^ = 46%; Higgins et al., 2003). The calculation of Rosenthal's “Fail-Safe N” (Rosenthal, 1979) indicates that it would take 149 studies with insignificant findings before the cumulative effect in the analysis would no longer be statistically significant (*p* > 0.05). Similarly, “Orwin's Fail-Safe N” (Orwin, 1983) suggests that in order to bring the criterion down to a trivial level, which represents an effect value other than zero (in this study defined as 0.05), 120 non-significant studies would be required. Given the total number of studies in this field (Figure 1), it appears unlikely that 149 or 120 studies, respectively that would yield non-significant or negative results, have not reached publication. The positive 95%-CI around the mean treatment effect (*d*) corroborates this conclusion further.

**Figure 3:**
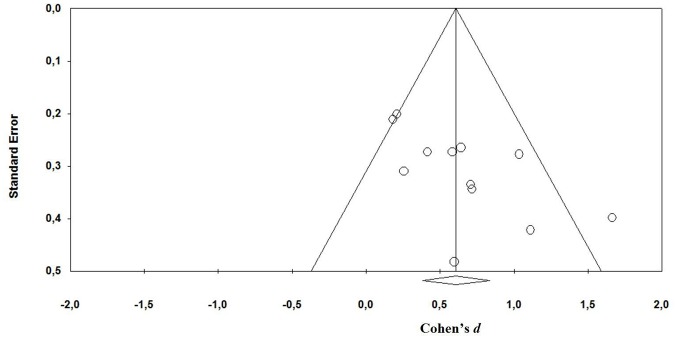
**Funnel plot illustrating proneness to publication bias for the included studies**. Mean effect size is *d* = 0.61 (CI 0.38, 0.84).)

Analysis of publication bias showed a fairly symmetrical funnel plot (Figure 3). Taken together, the calculated overall effect size seems to be rather robust and not to be strongly affected by a publication bias.

### Subgroup analysis

Overall effect sizes were separately analyzed for personalized vs. group intervention, individualized vs. prescribed music and active vs. passive intervention (Table 2). Both personalized and group intervention showed comparable substantial effect sizes (*d*_*diff*_ = 0.14). Results of personalized intervention showed larger heterogeneity indicating lower comparability of results.

**Table 2:** **Subgroup differentiation**.

**Methods**	**Number of studies**	**Cohen's *d* (95% CI)**	**Heterogeneity**	
			***I*^2^ (%)**	***P***
Group therapy	7	0.53 (0.33–0.74)	11.10	0.345
Personal therapy	6	0.68 (0.18–1.17)	67.87	0.008
Individualized music	9	0.54 (0.32–0.77)	28.37	0.192
Prescribed music	2	0.49 (0.09–0.88)	0.00	0.329
Active music intervention	8	0.61 (0.41–0.81)	0.00	0.51
Passive music intervention	6	0.65 (0.17–1.13)	70.73	0.004
Moderate to severe dementia	11	0.63 (0.38–0.89)	50.22	0.028
Mild to moderate dementia	1	0.42 (0.95–0.11)		

*CI, confidence interval*.

Both individualized and prescribed music showed a medium effect size (*d*_*diff*_ = 0.05). However, because only two studies used prescribed music compared to individualized music with nine studies it is difficult to draw a conclusion from these results that can be generalized to the population.

Active and passive interventions yielded nearly identical effect sizes (*d*_*diff*_ = 0.04). This comparison has to be interpreted with caution as passive MI intervention seem to be nearly too heterogeneous to be analyzed as a category. The category of active MI interventions, however, consisted of a highly homogeneous group of studies.

## Discussion

The systematic evaluation of effects of music intervention is a prerequisite for the systematic improvement of these interventional approaches (Vink et al., 2011). This study evaluated the effectiveness of music intervention on agitation in demented people. Results suggest a stable medium positive effect. Effects of controlled studies varied between *d* = 0.18, 95% CI, −0.23–0.60 (min) and *d* = 1.11, 95% CI, 0.29–1.94 (max) (mean *d* = 0.61, 95% CI 0.38–0.84). The effect size will be considered clinically significant by most practitioners and the therapy is furthermore appealing because of its low risk profile and moderate cost. Individual MI showed tendentially higher effects than MI applied in groups, but much larger variations of these effects. This might indicate the particular importance of so far underreported influencing factors contributing to the success of individual MI settings, but can also be related to the larger heterogeneity of included studies, their settings, designs and target groups. The subgroup analysis of individualized and prescribed MI showed both a medium effect. With the two included studies on prescribed music, the results turned out to be highly homogeneous. Individualized MI consisted of a larger group of studies, and showed a small, but yet heterogeneous result. While active and passive MI yielded very similar mean effects, passive interventions showed remarkably higher heterogeneity between studies and consequentially larger variations of effects. Further research is needed to investigate the predictors of successful MI in regards to the particular settings before conclusions on the underlying causes for these differences can be drawn.

### Limitations

While the overall effect of MI can be considered to be robust with effect sizes ranging from 0.18 to 1.11, the subgroup analysis suffers from the low number of studies providing sufficient statistical information to be included in this meta-analysis, and from high heterogeneity within the group of personalized MI and passive MI. Furthermore, this meta-analysis did not differentiate between various types of dementia or degrees of severeness. This information is usually not provided and could account for varying effect sizes between studies. Diagnosis of dementia includes a wide range of symptoms, stages of progression, comorbidities and effects of pharmaceutical treatment. The majority of research investigated MI effects in patients with moderate to severe dementia. Patients with severe dementia have reduced abilities of emotional expression (Sung et al., 2006b; Sakamoto et al., 2013). Which type of MI is more or less suitable for different types of dementia and degrees of severeness can't yet be answered due to the relative early stage of systematic research in this field.

### Further research

For further studies evaluating the effectiveness of MI we recommend to differentiate between diagnoses, clinical samples and various degrees of severity, where this is possible due to the limited amount of research in this field. Future studies should also be careful in describing the actual setting of intervention (e.g., group size) so that future reviews and meta-analyses can investigate additional contributing and differentiating factors, conclude on underlying causes of the MI effect and propose modifications to maximize intervention effects.

Agitation is a frequent problem among demented patients increasing the caregivers' burden, impairing the quality of social interaction with other persons, it deteriorates with age (Cerejeira et al., 2012) and can therefore be of particular relevance. However, other aspects of BPSD might be affected as well and deserve additional attention in future research, once a sufficiently large body of original research can be provided.

The included studies also have implications for the time at which music intervention may be most effectively administered. Janata (2012) reported lower agitated behaviors and the frequency of this behavior for the morning observations than for the afternoon observations using the Cohen-Mansfield Agitation Inventory. The fact that the frequency of agitated behavior appears to be partially dependent on the time of day should be considered when measuring agitation in dementia and planning musical interventions. Another research question pending further systematic investigation is in how far MI effects persons in contact with the patients which are not subject to the intervention themselves (health care staff and relatives, other patients). A recent study reported that participants in the MI intervention group were less agitated and anxious after the sessions (Sung et al., 2012), and were thus less likely to evoke these emotions in other residents, including control group participants, in the care facility (Sung et al., 2012). While this situation was previously considered a limitation in a particular study, it can be hypothesized that MI facilitates positive emotions and well-being for those with indirect contact with music interventions. These secondary effects should be considered in more future study designs. The experimental group in Raglio et al. (2010) study received three cycles of treatment followed by 1 month of washout period after each cycle. The results from this intervention demonstrated that music intervention is an efficacious intervention even though the treatment was interrupted for 1 month. In the overall literature of music intervention and dementia, there is lacking evidence of long-term effects and its effectiveness of therapy. The Raglio et al. (2010) study provides interesting contributions to the reduction of agitation, indicating that the length of treatment may not matter significantly, as long as the treatment is available and sustained. However, for future research, longer follow-up assessments to determine possible beneficial effects of music intervention are recommended.

This meta-analysis focused on agitation as a major aspect of problematic behavior in dementia. Other outcome measures such as quality of life, depression and cognitive functions might also be of interest but are currently underrepresented or do not yet allow for comparison due to insufficient comparability of methods and designs. Previous studies documented positive effects of MI on depression (Guétin et al., 2009; Janata, 2012; Raglio et al., 2015), anxiety (Sung et al., 2012), cognitive function, and quality of life (Cooke et al., 2010; Raglio et al., 2015). However, more quantitative data is needed in randomized controlled studies for computing more accurate effect sizes in depression, cognition, anxiety, and quality of life. For a review of the effectiveness music intervention has on these outcomes it is recommended to consult the meta-analyses of Vasionyte and Madison (2013), and Ueda et al. (2013). Vasionyte and Madison (2013) did not have any particular inclusion criteria in the study design of their meta-analysis. The results of Vasionyte and Madison's (2013) indicated that music therapy significantly improved cognitive functioning in demented people, reflecting a high effect size. Ueda et al. (2013) included RCTs, controlled clinical trial and controlled trials in their meta-analysis. Their results revealed that music interventions reduced depression to a small extent, and the results of anxiety reflected a moderate effect size.

## Conclusion

This meta-analysis of music interventions for demented people showing agitation provides evidence for the effectiveness of music intervention in treatment of agitation in dementia. The analysis validates a non-pharmacological approach in treatment of agitation, a particular detrimental symptom of dementia. The overall medium effect size of this meta-analysis suggests that music intervention can reduce agitation in persons with dementia.

## Author contributions

SP collected the data material and was in charge of drafting the manuscript. PA, RL, MA, and SS wrote parts of the manuscript, proofread and revised, and provided guidance through all stages.

### Conflict of interest statement

The authors declare that the research was conducted in the absence of any commercial or financial relationships that could be construed as a potential conflict of interest.
